# Editorial

**Published:** 1875-07

**Authors:** 


### Editorial.

The remarks of Prof. Gross, at the late meeting of the American Medical Association, appear to have revived the blood-letting controversy, and the sharpening up of old time worn lancets has become a new professional industry in the hands of many emulous of the great surgeon's fame, but totally unappreciative of the true intent of his remarks, which was to deprecate the total abolition from therapeutics of that which, under some circumstances, judiciously used, might prove a valuable agent; and by no means originate a new brood of Sangrados, whose panacea should be depletion.

As a compensating agent for the maintenance of the circulatory balance, in danger of disturbance by these "leeches," comes another Nestor—Prof. Freer—to the front, with his transfusion tube, and defibrinating twigs, and shows not only that a liberal supply of good defibrinated blood is necessary to the life, but, better still, demonstrates the practicability and facility of supplying deficiencies in the amount of this fluid in the vessels of the human economy, occasioned by accident, disease, or the Sangrados of the future.

But, as a further reminder of the old adage, "Blood will tell," comes Dr. J. G. Richardson, of Philadelphia, with his "high powers," and demonstrates to a Syracuse jury, that the blood of a murdered man, clinging to the sleigh of his assassin, will cry out for vengeance, even though drenched in the blood of a hog subsequently slaughtered upon the same sleigh, and washed with hot water.

Dr. Richardson's opinions upon this subject, embodied in his paper on blood stains, published in the Am. Jour. Med. Sciences for July, 1874, are sharply contested by Dr. J. J. Woodward, U. S. A., another microscopical authority, in a very carefully written article in the Jan. No. of the Am. Jour. Med. Sciences, which, as a contribution to science, is unimpeachable, demonstrating the extreme difficulty, if not impossibility, of discriminating between the blood corpuscles of man and many other mammalia, *ex. gr.*, Guinea-pigs, monkeys, seals, otters, kangaroos, capybaras, wombats, porpoises, and, more especially, dogs.

But, while the paper is faultless in itself, when compared with that which it criticizes, it looks a little like special pleading, inasmuch as comparisons are made with the blood of a different series of animals from those enumerated in the original paper. This, however, is not the most important feature in the discussion. For while it is true, as Dr. Woodward says, that we can "never affirm truthfully, on the strength of microscopical investigation, that a given stain is positively composed of human blood," which affirmation Dr. Richardson endorses, it is nevertheless equally true, that it rarely happens that microscopical investigation alone is required to decide such questions, but only to constitute one link in a long chain of testimony to which it is merely supplementary. That under these circumstances an affirmative answer can be given to the question of the differential diagnosis between human blood and that of certain of the mammalia, appears to be demonstrated by Dr. Richardson's recent measurements.

A further question between these two distinguished observers appears to have arisen, as to the extent to which an expert on the witness stand is allowed to go in his explanations to a jury. Usually it will be found that he will not be permitted by counsel to transcend the limits of the facts in evidence, and their relations; and when the question is to describe the differences between the blood of a human being and that of a hog, he will not be allowed to introduce comparisons with that of dogs or other animals. H.

*Mr. Editor*—A question, if you please. Given, a healthy primapara, sick thirteen hours, in a natural twin labor: could you account for her death occurring a very short time after the delivery of the last child, provided you knew she took chloroform estimated at from one to four pounds, and the second child not delivered for four hours after the first, patient complaining of exhaustion constantly?

Dr. Mesentery, the sleepy tub who waited on her, explains it, that as the labor was a dry one, the waters that should have discharged in the natural way overflowed her lungs, and drowned her. What say ye of him?

It is much easier to ask questions than to answer them. We have heard of deaths resulting from the use of chloroform, and should much prefer that solution of the problem, to the one offered by "Dr. Mesentery," viz., "that the waters which should have discharged in the natural way overflowed her lungs, and drowned her." Not being an anatomical expert, we are puzzled to know how the water could get there, *i. e.*, to "the lungs;" and, further, if this be the true reason, it suggests a danger to which "Dr. Mesentery" himself might be liable, in case he should assume the horizontal position, *i. e.*, that the vesical contents might overflow his brains and drown him.—Ed.

During the past year, certain experts in neurology, residing in New York City, impressed with the belief that their special field of medical science could be more successfully cultivated by thoroughly systematizing the work, and by uniting the laborers therein under one organization, conceived a plan for the formation of an association which should embrace a large number of workers in the department of neurology in different parts of the world, who, by co-operative investigation, and periodical reunion for report and conference, might afford mutual aid and instruction, and place the science upon a substantial basis of fixed principles. For the purpose of preliminary organization, a committee was selected, consisting of Drs. W. A. Hammond, Meredith Clymer, and E. C. Seguin, who, upon consultation, invited a number of gentlemen identified with the study of neurology in different sections of the United States, to meet in the city of New York, on the 2d day of June, 1875. The invitation was accepted, with very few exceptions, by all invited.

The first meeting was held upon the day specified, and an organization was speedily effected upon an admirable plan, carefully prepared in advance by the committee previously indicated. So well had this work been accomplished that no serious objection was made to its acceptance, and, upon its adoption, the American Neurological Association became a fixed fact, with the following list of members, viz. : Dr. Schmidt, of New Orleans ; Dr. Bauduy, of St. Louis ; Dr. Roberts Bartholow, of Cincinnati ; Drs. J. S. Jewell, Walter Hay, and H. M. Bannister, of Chicago ; Drs. Miles, and Van Bibber, of Baltimore ; Drs. S. Weir Mitchell, H. C. Wood, and W. Pepper, of Philadelphia ; Dr. Hunn, of Albany ; Dr. Lente, of Cold Spring, N. Y. ; Drs. Weber, J. J. Putnam, Lincoln, and Clarke, of Boston ; Drs. W. A. Hammond, Meredith Clymer, E. C. Seguin, T. M. B. Cross, J. J. Mason, Allan McLane Hamilton, J. M. Beard, Rockwell, Arnold, Wagner, and McBride, of New York.

The following officers were elected unanimously, to serve until the next annual meeting, viz.:

President, Dr. J. S. Jewell, of Chicago.

1st Vice-President, Dr. F. T. Miles, of Baltimore.

2nd Vice-President, Dr. Clarke, of Boston.

Recording Secretary and Treasurer, Dr. E. C. Seguin, New York.

Corresponding Sec'y, Dr. Jno. J. Mason, New York.

Curator, Dr. Arnold, New York.

The membership is limited by the Constitution to fifty residents of the United States, and twenty-five Foreign Corresponding Members. Candidates to be nominated by one member, endorsed by two others, and to present a paper, which shall be referred to the Executive Committee for criticism, and to be voted for at the next annual meeting. Nineteen papers were presented, of which thirteen were read; the remainder have been omitted solely for want of time to read, the hour for final adjournment having arrived. These will, however, all be published in the first Vol. of the Transactions of the American Neurological Association, which will appear early in the course of the current year.

The meeting of the Association was characterized by a remarkable degree of harmony and unanimity; it was organized for work, and accomplished its mission faithfully. The uniformity of work, however, was varied by the proverbial elegant hospitality of the New York members, who entertained their associates in a style and manner unattainable outside the metropolitan city. The thanks of every student of neurological science in the United States are especially due to Drs. Clymer, Hammond and Seguin, for organizing a nucleus around which the neurological science of the future must gather.

H.

***Explanation.***

The June No. of the JOURNAL was complete as to matter before the departure of the managing Editor to attend the meeting of the American Neurological Association in New York. But "the best laid schemes of mice and men gang aft aglee." During his absence and before the matter was sent to press it became most marvellously deranged. The microscopic investigation of Dr. J. G. Richardson, of Philadelphia, which was a part of a report of a case of Traumatic Tetanus and an important supplement thereto, was published as an independent article, No. 1, whereas the report which it was intended to supplement, was published as article No. 3, without any head at all.

Article No. 2, "Hereditary Transmission, etc.," was likewise decapitated instead of being graced with the name of Dr. L. C. Mitchell, its author, by whom it was read before the Will County Medical Society.

We are not habitually profane, but we thought a very large d—— nevertheless, but consoled ourselves with an amendment to the old nursery rhyme, "when the cat is away the mice will play"—the devil. H.

### *Prize Essay—Medical Association State of Alabama.*

At the Annual Session of this Association, held at Montgomery during the second week in April, 1875, Dr. S. D. SEELYE, of Montgomery, after some remarks regarding the increasing frequency with which cases of Bright's Disease are brought under medical care in this section, the unsatisfactory though extensive literature of the subject, and the generally fatal prognosis which we are obliged to give, rendering it one of the opprobia of the profession; and believing that there is still some hidden light that may be made to illumine the subject if attention and inquiry are turned especially to it; proposed to place in the hands of this Association a prize of one hundred dollars for the best treatise on this disease, under the following regulations, viz.:

1st. Competition for the prize to be open to the whole country.

2nd. A committee of five to be appointed to adjudicate upon the essays presented.

3rd. All essays to be forwarded to the chairman of said committee on or before the first day of February, 1876, and to be accompanied by a sealed letter containing name and address of the author, which letter shall not be opened until after the adjudication is made.

4th. The Prize Essay to be the property of this Association, and to be published in its Annual Volume of Transactions; and all unsuccessful papers to be returned to the address of the authors; honorable mention being made of any deemed of especial merit.

5th. If none of the essays presented are deemed worthy of the prize, the committee shall have the privilege of rejecting all, in which case the competition shall be open for another year.

6th. The prize shall be one hundred dollars in currency, with a certificate of this Association suitably inscribed and bearing the seal of the Association; or it will be wrought into a Gold Medal or Plate with a suitable legend and a fac simile of the seal of the Association engraved thereon, to be of the full value of one hundred dollars, less the price of manufacture, at the option of the successful author.

7th. The adjudication shall be publicly announced at the next Annual Meeting of the Association, to be held at Mobile during the second week in April, 1876.

The above proposition having been accepted by this Association, it now invites from the thinkers and investigators of the profession at large the generous competition which its author invokes.

*Committee of Adjudication.*—Dr. Jerome Cochrane, Mobile, Ala., *Chairman;* Dr. J. B. Gaston, Montgomery, Ala.; Dr. W. H. Anderson, Mobile, Ala.; Dr. Peter Bryce, Tuscaloosa, Ala.

BENJ. H. RIGGS, M.D.,

Sec. M. A. S. A., Selma, Ala.

